# Biodiversity of testate amoebae in *Sphagnum* bogs: the dataset from forest-steppe ecotone (Middle Volga Territory, Russia)

**DOI:** 10.3897/BDJ.12.e125582

**Published:** 2024-06-12

**Authors:** Nailia M. Saldaeva, Kirill V. Babeshko, Viktor A. Chernyshov, Anton S. Esaulov, Alexander A. Komarov, Nikita R. Kriuchkov, Natalia G. Mazei, Damir A. Saldaev, Tamara G. Stojko, Andrey N. Tsyganov, Yuri A. Mazei

**Affiliations:** 1 Shenzhen MSU-BIT University, Shenzhen, China Shenzhen MSU-BIT University Shenzhen China; 2 Lomonosov Moscow State University, Moscow, Russia Lomonosov Moscow State University Moscow Russia; 3 Penza State University, Penza, Russia Penza State University Penza Russia

**Keywords:** the Volga Upland, peatlands, Arcellinida, Rhizaria

## Abstract

**Background:**

Testate amoebae are a polyphyletic group of unicellular eukaryotic organisms that are characterised by a rigid shell and inhabit mostly freshwater and terrestrial ecosystems. They are particularly abundant in peatlands, especially in *Sphagnum*-dominated biotopes. Peatland hydrology is the most important influence on testate amoebae communities. The good preservation of the shells in peat deposits and their response to hydrological regime changes are the principles for palaeohydrological reconstructions. Any changes in the water balance of mires should be expected to have far-reaching effects on biogeochemical cycles, productivity, carbon dioxide and methane exchange.

**New information:**

This paper presents a dataset (Darwin Core Archive – DwC-A) on the distribution of *Sphagnum*-dwelling testate amoebae in nine mires located in the forest-steppe subzone of the East European Plane. The dataset includes information about 86 taxa belonging to 29 genera and contains 3,123 occurrences of 49,874 individuals. The following environmental variables are provided: microtopography, oxidising and reducing potential, total mineralisation, substrate temperature, acidity, substrate wetness and water table depth. These data might be used for biogeographical and palaeoecological studies, including quantitative reconstructions.

## Introduction

Testate amoebae are eukaryotic unicellular organisms that are enclosed in a rigid cover called a shell or a test. The shell has one or two openings (pseudostome) through which filose or lobose pseudopodia protrude during locomotion and feeding (Cavalier-Smith 2004). It is a polyphyletic group within Supergroup Amoebozoa (Adl et al. 2019) related to Phylum Tubulinea (Smirnov et al. 2005) and Supergroup Sar related to Phylum Stramenopiles (Adl et al. 2005) and(?) Rhizaria (Cavalier-Smith 2004). Testate amoebae are particularly abundant in peatlands, where they can constitute up to 50% of microbial biomass (Gilbert and Mitchell 2006). Thus, studying the biodiversity of these organisms provides an important contribution to understanding the structure and functional role of microbial communities.

Testate amoebae are widely used in bioindication (Payne 2007) of water table depth and surface moisture in mires (Zheng et al. 2019, Krashevska et al. 2020, Swindles et al. 2020, Qin et al. 2021b, Šímová et al. 2022), organic matter content (Laggoun‐Défarge et al. 2007, Escobar et al. 2008, Daza et al. 2020), macronutrients (Wilkinson and Mitchell 2010, Patterson et al. 2012a), acidity (Payne 2009, Lamentowicz et al. 2011, Patterson et al. 2012b, Siver et al. 2020, Sim et al. 2021), metal ions concentration (Nguyen-Viet et al. 2006, Asada and Warner 2009, Cockburn et al. 2020) in aquatic environments, ecosystem restoration (Carballeira and Pontevedra-Pombal 2021, Qin et al. 2021a, Evans et al. 2023, Creevy et al. 2023) etc. Testate amoebae are successfully applied in quantitative reconstructions of hydrological regimes (Lamentowicz et al. 2017, Tsyganov et al. 2017, Swindles et al. 2019, Zhang et al. 2022), serving as a valuable proxy.

The growing number of available resources on these microorganisms allows for solving large-scale issues of biogeography and problems of flagship, endemic and eurybiotic species (Foissner 2006). The dataset presented in this paper represents the results of five years of investigations on testate amoebae in *Sphagnum*-dominated bogs of its southern boundary distribution in the forest-steppe ecotone (Tsyganov and Mazei 2007, Mazei and Tsyganov 2007a, Mazei and Tsyganov 2007b, Mazei et al. 2007a, Mazei et al. 2007b, Mazei and Bubnova 2007, Mazei and Bubnova 2008, Mazei and Bubnova 2009, Mazei et al. 2009, Tsyganov et al. 2016).

## Project description

### Design description

The description of each observation in the dataset is based on terms used in the general Darwin Core vocabulary. In the dataset, each observation includes basic information on the location (latitude and longitude), date of observation, name of the observer and number of counted individuals. The coordinates were determined in situ using a GPS device. For mire ecosystems, sampling locations contain information on microtopography (hummocks, lawns, hollows or not available), oxidising and reducing potential (redox), total mineralisation (tds), substrate temperature, acidity (pH), substrate wetness and mire water table depth (WTD) (Table 3).

## Sampling methods

### Sampling description

Samples were generally collected in the biotopes dominated by *Sphagnum* spp. mosses and less frequently by *Polytrichum* spp. The sampling strategy tried to cover all the diversity of the microtopography of the mires (hummocks, lawns and hollows). Mosses were carefully extracted from the moss carpet and cut into layers according to the vertical zonation of peat soils: first from 0 to 15 cm by a 3 cm step and then the rest of the entire part of the dead mosses (Dobrovol’skii et al. 1998). After that, samples were placed in plastic containers and fixed with a formaldehyde solution in situ to avoid major post-sampling changes in the community structure (Mazei et al. 2015). Additional samples were taken for moisture content measurements. Water table depth (cm) was measured at each sampling point in a hole in relation to water on the surface of the moss cover after at least 30 minutes. Oxidising and reducing potential, total mineralisation, substrate temperature and pH value were measured using portable HANNA multiparameter meters in situ.

In the laboratory, samples were thoroughly shaken and stirred for 10 minutes in distilled water to extract testate amoebae. The suspension without *Sphagnum* stems was poured off to a Petri dish; live amoebae and empty tests were identified and counted separately in one-tenth part of the entire Petri dish using a stereomicroscope at 65× magnification. If necessary, the shells were transferred to a slide with a thin pipette, placed in a drop of glycerol and investigated at 150× or 300× magnification using a light microscope. A minimum count of 300 shells in each sample was achieved. The taxonomic classification at the genus level is based on the revisions of Kosakyan et al. (2016) as summarised in Tsyganov et al. (2016), González-Miguéns et al. (2021) and González-Miguéns et al. (2022). Moisture content was determined from additional samples taken in the field. Wet samples were weighed and placed in an oven at 105°C for eight hours. The samples were then cooled in a desiccator to room temperature and then weighed again. Percentage moisture was calculated, based on the difference between the wet and dry sample weights.

## Geographic coverage

### Description

**Figure 1.**
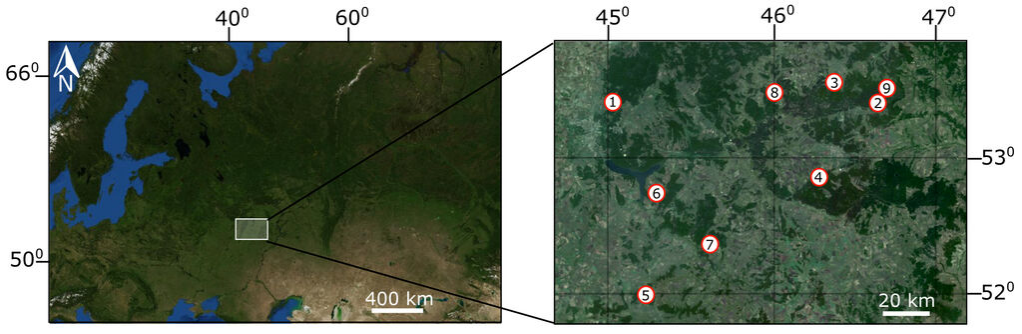
Map of the sampling sites. The numbers in the circles are mires, 1 – Bezymianoe; 2 – Svetloe; 3 – Kachim; 4 – Verkhozimskoe; 5 – Chibirley; 6 – Naskaftiym; 7 – Ivanovskoe; 8 – Sosnovoborsk; 9 – Kuncherovo.

The investigations were conducted from 2004 to 2006 in the Penza Region, Russia. The Penza Region is located in the west of the Volga Upland on the East European plain. It belongs to the forest-steppe subzone and the climate is temperate continental. The average annual air temperature in 2004–2006 is 5.8°C; precipitation is 627 mm (Weather and climate 2023). Nine mire ecosystems are included in this dataset (Fig. 1).

Bezymianoe mire (53.30463°N, 45.13816°E) was sampled once a month from 20 May to 26 September 2004. The bog is circular and about 300 m in diameter. The vegetation of lawns is dominated by *Calamagrostiscanescens* (Weber) Roth., *Eriophorumvaginatum* L. and *Menyanthestrifoliata* (L.). The centre of the mire is overgrown with *Betulapubescens* Ehrh. and *Pinussylvestris* L., together with the shrub *Myrtuscommunis* L. The moss cover is flat, with the predominant species *Sphagnumpalustre* L., *S.divinum* Flatberg & K. Hassel and *S.angustifolium* (C.E.O. Jensen ex Russow) C.E.O. Jensen. The hummocks in the middle of the mire are formed by *S.papillosum* Lindb. and *S.angustifolium*, *Polytrichumstrictum* Brid. and *Droserarotundifolia* L. Due to peat excavation, there is a drain channel at the edges of the mire and several ditches with open water in the centre, where *Utriculariavulgaris* L. and *Sparganiumminimum* Wallr. were common. The edge of the *Sphagnum* quagmire is formed by *S.riparium* Ångstr.

Svetloe mire (53.33073°N, 46.82112°E) was sampled on 3 and 27 June 2004. It represents an overgrowing *Sphagnum* mat around the Svetloe Lake (the area is 7.2 ha). The lake shore is surrounded by reed vegetation composed of *Calamagrostiscanescens* (up to 90%), *Phragmitesaustralis* (Cav.) Trin. ex Steud., *Carexriparia* Curtis and a small amount of *Betulapendula* Roth. and *Salix* sp. Samples for testate amoebae were only collected in a *Sphagnum*-dominated mat with the presence of *Pteridiumaquilinum* (L.) Kuhn, *Melampyrumnemorosum* L., *Polygonatumodoratum* (Mill.) Druce and *Vacciniummyrtillus* L.

Kachim mire (53.36187°N, 46.58564°E) was sampled on 27 June 2004. This is the largest oligotrophic mire in the region, with an area of 39.2 ha. The mire is round and surrounded by a drainage channel. Various *Sphagnum* mosses dominate (up to 70%) the vegetation cover, with *E.vaginatum* growing on hummocks. The tree cover is represented by the rare species *B.pubescens* and *P.sylvestris*. *Andromedapolifolia* L., *Oxycoccuspalustris* Pers. and *Droserarotundifolia* are occasionally found as well. The abundance of *Carex* spp., *Comarumpalustre* L. and *Naumburgiathyrsiflora* (L.) Rchb. increases at the edge of the mire.

Verkhozimskoe mire (52.98561°N, 46.45928°E) was sampled on 28 June 2004. It represents a mire complex with a total area of 8.1 ha, separated by a drainage channel. Mires are covered with various *Sphagnum* and *Polytrichum* species. The hummocks in the central part are formed by *Carexlasiocarpa* Ehrh., *C.vesicaria* L., *Eriophorumvaginatum* and *Droserarotundifolia*. The other grasses that might be observed in the mire are *C.riparia*, *C.cinerea* Poll., *Carexomskiana* Meinsh., *Moliniacaerulea* (L.) Moench, *Comarumpalustre*, *Menyanthestrifoliata*, *Lysimachiavulgaris* L., *Naumburgiathyrsiflora* and *Galiumpalustre* L., *Pinussylvestris*, *Betulapubescens* and *Myrtuscommunis* are found sporadically. *Utriculariavulgaris* is found only in waterlogged drainage channels.

Chibirley mire (52.91076°N, 46.62264°E) was sampled in June 2004. The mire is covered predominantly by *Sphagnum* mosses (*Sph.riparium*, *Sph.centrale* C.E.O.Jensen, *Sph.palustre* and *Sph.capillifolium* (Ehrh.) Hedw.) and, less abundantly, by diverse *Polytrichum* species. There are *Betulapubescens*, *B.humilis* Schrank, *Pinussylvestris*, *Oxycoccuspalustris* and *Chamaedaphnecalyculata* (L.) Moench. in the central part of the mire. *Droserarotundifolia* was found on hummocks. Amongst the grasses, *Carexrostrata* Stokes, *C.cinerea* and *Eriophorumpolystachyon* L. were observed. At the edge of the mire, the vegetation was formed by *Calamagrostiscanescens*, *Eriophorumvaginatum*, *Comarumpalustre*, *Lysimachiathyrsiflora*, *Typhalatifolia* L. and *Menyanthestrifoliata*. *Salixcinerea* and *S.aurita* L. were found sporadically.

Naskaftiym mire (52.93960°N, 45.47969°E) was sampled on 15 July 2004. It is a round bog (10 ha) that is completely covered by *Sphagnum* mosses (*Sph.centrale*, *Sph.fallax* (H.Klinggr.) H.Klinggr., *Sph.flexuosum* Dozy & Molk., *Sph.girgensohnii* Russow and *Sph.obtusum* Warnst.). The surface of the mire is relatively flat. Grasses are represented by *Carexlimosa* L., *C.cespitosa* L., *C.hartmaniorum* Cajander, *C.rostrata*, *Eriophorumangustifolium* Honck., *E.gracile* W.D.J. Koch, *E.vaginatum*, *Calamagrostiscanescens* and *Phragmitesaustralis*. *Comarumpalustre*, *Equisetumfluviatile* L., *Peucedanumpalustre* (L.) Moench. and *Menyanthestrifoliata* are also found. Shrub *Salixlapponum* L. and trees of *Betulapubescens* are very rare.

Ivanovskoe mire (52.70788°N, 45.82308°E) was sampled on 28 July 2004. It represents a lake that is formed as a result of peat excavations in a mire and is overgrown by a *Sphagnum*-dominated mat. The area of the mire is 25 ha. The following types of mosses are found: *Sphagnumriparium*, *Sph.squarrosum* Crome and *Sph.papillosum*. The other mat-forming plants are *Comarumpalustre*, *Menyanthestrifoliata*, *Carex* spp., *Typhalatifolia*, *Phragmitesaustralis*, *Betulapubescens* and *Salixcinerea*.

Sosnovoborsk mire (53.31500°N, 46.19544°E) was sampled on 5 June 2005. It represents a waterlogged pine forest with shallow peat deposits. The tree cover is generally composed of *Pinussylvestris* with an admixture of *Betulapubescens*, *Frangulaalnus* Mill., *Sorbusaucuparia* L. and *Chamaecytisusruthenicus* (Fisch. ex Woloszcz.) Klásk. The shrubs and herbs are *Vacciniummyrtillus*, *Vacciniumvitis-idaea* L. and *Rubussaxatilis* L. The ground cover is formed by *Lycopodiumannotinum* L., *L.clavatum* L., *Diphasisastrumcomplanatum* (L.) Holub., *Luzulapilosa* (L.) Willd. and *Pteridiumaquilinum*. Mosses are *Polytrichumcommune* Hedw., *Sphagnumdenticulatum* Brid., *Sph.girgensohnii*, *Sph.centrale*, *Sph.russowii* Warnst. and *Sph.divinum*.

Kuncherovo mire (53.34832°N, 46.83608°E) was sampled on 4 July 2006. This is a round in shape, mesotrophic mire with an area of 2 ha. At the edges, *Salix* sp. and hummocks formed by *Carexcanescens* L. were found. The central part is composed of *Betulapubescens*, *Eriophorumvaginatum* and a well-developed *Sphagnum* moss cover. *Scheuchzeriapalustris* L., *Comarumpalustre* and *Menyanthestrifoliata* are rare. *Sphagnum* moss cover is composed of *Sph.divinum*, *Sph.angustifolium* and *Sph.squarrosum*.

### Coordinates

52.70788°N and 53.36187°N Latitude; 45.13816°E and 46.83608°E Longitude.

## Taxonomic coverage

### Description

The dataset represents information on the distribution of 86 species of testate amoebae in *Sphagnum*-dominated bogs in the forest-steppe ecotone. There are a total of 29 genera, which belong to 16 families and three incertae sedis ranks (Table 1). In total, 49,238 individuals were identified with 3,123 occurrences (Kriuchkov et al. 2023). The greatest number of genera were in the families Hyalospheniidae (4) and incertae sedis (3), including *Argynnia*, *Physochila* and *Trigonopyxis*. Families Arcellidae, Assulinidae, Centropyxidae, Euglyphidae, Netzeliidae and Trinematidae include two genera and all the others contain only one. The largest number of taxa were found in Arcellidae (18), Difflugiidae (11) and Euglyphidae (11).

The most abundant species in the dataset (Table 2) are *Assulinamuscorum* (11.9% of the total number of counted individuals), which also has the highest occurrence (281). The species *Hyalospheniapapilio* (11.7%) is almost equally abundant as the previous one, whereas the other species had lower abundances: *Physochilatenella* (7.5%), *Galeriporaarenaria* (7.3%), *Nebelatincta* (7.0%), *Heleoperasphagni* (6.8%), *Phryganellahemisphaerica* (6.7%), *Hyalospheniaelegans* (5.7%), *Assulinaseminulum* (5.0%), *Euglyphaciliata* (3.9%), *Euglyphalaevis* (2.5%), *Galeriporacatinus* (2.4%), *Archerellaflavum* (2.1%), *Centropyxisaculeata* (2.0%), *Difflugiaparva* (1.1%) and Heleoperapetricola (1.1%). A total of 20 taxa are less abundant than 0.02% and are assumed to be rare: *Lesquereusiaspiralis*, *Euglyphacristatadecora*, *Sphenoderiafissirostris*, *Galeriporapolypora*, *Difflugiaurceolata*, *Pseudodifflugiagracilis*, *Difflugiaoblonga*, *Galeriporamegastoma*, *Difflugiapyriformis*, *Scutiglyphascutigera*, *Argynniadentistoma*, *Centropyxisecornis*, *Euglyphaacanthophora*, *Euglyphastrigosaglabra*, *Placocistaglabra*, *Centropyxisconstricta*, *Cyclopyxisaplanatamicrostoma*, *Difflugiabacillifera*, *Placocistalens*, *Difflugiabrevicolla*, *Arcellavulgarisundulata*, *Arcellavulgarispenardi* and *Lesquereusiainequalis*. The following species were found in more than a third part of all sample sets: *A.muscorum* (281), *N.tincta* (186), *H.papilio* (185), *A.seminulum* (176), *E.ciliata* (173), *H.elegans* (136), *E.laevis* (131), *P.tenella* (130), *G.arenaria* (127), *H.sphagni* (124), *P.hemisphaerica* (118), *A.flavum* (109) and *C.aculeata* (103). There are 12 species that were observed only once: *Pseudodifflugiagracilis*, *Galeriporamegastoma*, *Scutiglyphascutigera*, *Argynniadentistoma*, *Euglyphastrigosaglabra*, *Cyclopyxisaplanatamicrostoma*, *Difflugiabacillifera*, *Placocistalens*, *Difflugiabrevicolla*, *Arcellavulgarisundulata*, *Arcellavulgarispenardi* and *Lesquereusiainequalis*.

## Usage licence

### Usage licence

Other

### IP rights notes

Creative Commons Attribution Non-Commercial (CC-BY-NC) 4.0 Licence

## Data resources

### Data package title

The dataset on terrestrial testate amoebae from forest-steppe ecotone (Middle Volga Territory, Russia) in 2004–2006.

### Resource link

https://www.gbif.org/dataset/66da1912-92fe-4527-95be-fb38b12b21a8

### Alternative identifiers

http://gbif.ru:8080/ipt/resource?r=penza&v=1.23 https://doi.org/10.15468/3d6gcr https://doi.org/10.15468/dl.4tu8q5

### Number of data sets

1

### Data set 1.

#### Data set name

The dataset on terrestrial testate amoebae from forest-steppe ecotone (Middle Volga Territory, Russia) in 2004–2006

#### Download URL

http://gbif.ru:8080/ipt/archive.do?r=penza

#### Description

The description of each observation in the dataset is based on terms used in the general Darwin Core vocabulary (GBIF.org 2023). In the dataset, each observation includes basic information on the location (latitude and longitude), date of observation, name of the observer and number of counted individuals. The coordinates were determined in situ using a GPS device. The dataset is structured using the Occurrences and Extended Measurements or Facts (eMoF) extension. The Extended Measurement or Fact table contains the fields listed in the table below. Sampling locations of mires contain information (i.e. measurementType) on microtopography (hummocks, lawns, hollows or not available), oxidising and reducing potential (redox), total mineralisation (tds), substrate temperature, acidity (pH), substrate wetness and mire water table depth (WTD).

**Data set 1.** 

Column label	Column description
eventID (Occurrence)	An identifier for the set of information associated with an Event.
parentEventID (Occurrence)	An identifier for the broad event of place and year.
samplingProtocol (Occurrence)	Descriptions of the methods and protocols used for material sampling.
samplingEffort (Occurrence)	The amount of effort expended during sampling procedure.
sampleSizeValue (Occurrence)	A numeric value for a measurement of the size (volume) of a sample.
sampleSizeUnit (Occurrence)	Cubic centimetre.
occurrenceID (Occurrence, eMoF)	An identifier for the occurrence (as opposed to a particular digital record of the occurrence).
eventDate (Occurrence)	The date when material was collected or sampling period.
basisOfRecord (Occurrence)	The specific nature of the data record.
kingdom (Occurrence)	The full scientific name of the Kingdom in which the taxon is classified.
scientificName (Occurrence)	The full scientific name, including the genus name and the lowest level of taxonomic rank with the authority.
habitat (Occurrence)	Notes about the dcterms:Location (microtopography, including hummocks, lawns and hollows).
family (Occurrence)	The full scientific name of the Family in which the taxon is classified.
class (Occurrence)	The full scientific name of the Class in which the taxon is classified.
taxonRank (Occurrence)	The taxonomic rank of the most specific name in the scientificName.
decimalLatitude (Occurrence)	The geographic latitude of location in decimal degrees.
decimalLongitude (Occurrence)	The geographic longitude of location in decimal degrees.
countryCode (Occurrence)	The standard code for the country in which the location is found, Russia (RU).
individualCount (Occurrence)	The number of individuals present at the time of the occurrence.
organismQuantity (Occurrence)	A number or enumeration value for the quantity of organisms.
organismQuantityType (Occurrence)	The type of quantification system used for the quantity of organisms (counted shells).
verbatimDepth (Occurrence)	The original description of the depth below the local surface (sampling depth from Sphagnum stems).
measurementType (eMoF)	The nature of the measurement, fact, characteristic or assertion (redox, total mineralisation, substrate temperature, pH, water table depth, substrate moisture).
measurementUnit (eMoF)	The units associated with the dwc:measurementValue (mV, mkSim/cm, °C, pH value, cm, %).
measurementValue (eMoF)	The value of the redox, total mineralisation, substrate temperature, pH, water table depth and substrate moisture measurement.
geodeticDatum (Occurrence)	WGS84
coordinateUncertaintyInMetres (Occurrence)	Coordinate uncertainty in metres (10).
coordinatePrecision (Occurrence)	A decimal representation of the precision of the coordinates (0.00001).
stateProvince (Occurrence)	The name of the next smaller administrative region than country (Penza Region).
minimumDepthInMetres (Occurrence)	The lesser depth of a range of depth below the local surface, in metres.
maximumDepthInMetres (Occurrence)	The greater depth of a range of depth below the local surface, in metres.
taxonRemarks (Occurrence)	Notes about the taxon valid name.
country	The name of the country or major administrative unit in which the Location occurs.

## Figures and Tables

**Table 1.** Species diversity of testate amoeba families in the dataset.

**Families**	**Number of genera**	**Number of taxa**	**Number of occu r rences**
Amphitremidae Poche, 1913	1	1	109
Arcellidae Ehrenberg, 1843	2	18	442
Assulinidae Lara et al., 2007	2	4	460
Centropyxidae Jung, 1942	2	7	244
Cryptodifflugiidae Jung, 1942	1	1	12
Difflugiidae Wallich, 1864	1	11	139
Euglyphidae Wallich, 1864, emend. Lara et al., 2007	2	11	398
Heleoperidae Jung, 1942	1	3	172
Hyalospheniidae Schultze, 1877 emend. Kosakyan and Lara, 2012	4	6	598
Incertae sedis (Class: Tubulinea)	3	4	188
Lesquereusiidae Jung, 1942	1	3	9
Microchlamyiidae Ogden, 1985, emend. Kudryavtsev et Hausmann, 2007	1	1	3
Netzeliidae Kosakyan et al., 2016, emend. Gonzales-Miguens et al., 2021	2	6	82
Phryganellidae Jung, 1942	1	2	148
Pseudodifflugiidae De Saedeleer, 1934	1	1	1
Sphenoderiidae Chatelain et al., 2013	2	3	11
Trinematidae Hoogenraad & De Groot, 1940, emend Adl et al., 2012	2	4	107
Total	29	86	3123

**Table 2.** Relative abundance (% to the total counts) and occurrences (samples) of testate amoebae forest-steppe ecotone (Middle Volga Territory, Russia). Species names are listed in the alphabetical order.

№	**Species name**	**Abundance**	**Occurrence**
1	*Alabastamilitaris* (Penard, 1890) Duckert, Blandenier, Kosakyan et Singer 2018	0.50	57
2	*Arcellaconica* Playfair, 1918	0.09	11
3	*Arcellagibbosa* Penard, 1890	0.35	20
4	*Arcellahemisphaerica* Perty, 1852	0.29	18
5	*Arcellaintermedia* (Deflandre, 1928) Tsyganov et Mazei, 2006	0.81	39
6	*Arcellamitrata* Leidy, 1876	0.16	17
7	*Arcellarotundata* Playfair, 1918	0.40	25
8	*Arcellavulgaris* Ehrenberg, 1830	0.27	19
9	*Arcellavulgarispenardi* Deflandre, 1928	0.00	1
10	*Arcellavulgarispolymorpha* Deflandre, 1928	0.97	9
11	*Arcellavulgarisundulata* Deflandre, 1928	0.00	1
12	*Archerellaflavum* (Archer, 1877) Loeblich et Tappan, 1961	2.07	109
13	*Argynniadentistoma* Penard, 1890	0.01	1
14	*Assulinamuscorum* Greeff, 1888	11.92	281
15	*Assulinaseminulum* Ehrenberg, 1848	4.90	176
16	*Bullinulariaindica* (Penard, 1907) Deflandre, 1953	0.52	56
17	*Centropyxisaculeata* (Ehrenberg, 1838) Stein, 1859	1.95	103
18	*Centropyxisaerophila* Deflandre, 1929	0.57	30
19	*Centropyxisaerophilasphangicola* Deflandre, 1929	0.79	45
20	*Centropyxisconstricta* (Ehrenberg, 1841) Penard, 1890	0.00	3
21	*Centropyxisecornis* (Ehrenberg, 1841) Leidy, 1879	0.01	4
22	*Centropyxisspinosa* Cash, 1905	0.15	3
23	*Corythiondubium* Taranek, 1871	0.53	31
24	*Cryptodifflugiacompressa* Penard,1902	0.81	12
25	*Cyclopyxisaplanatamicrostoma* Schönborn, 1966	0.00	1
26	*Cyclopyxisarcelloides* (Penard, 1902) Deflandre, 1929	0.22	11
27	*Cyclopyxiseurystoma* Deflandre, 1929	0.93	49
28	*Cyclopyxiskahli* (Deflandre, 1929)	0.07	8
29	*Difflugiabacillifera* Penard, 1890	0.00	1
30	*Difflugiabrevicolla* Cash et Hopkinson, 1909	0.00	1
31	*Difflugiaglans* Penard, 1902	0.05	6
32	*Difflugiaglobulosa* Dujardin, 1837	0.68	26
33	*Difflugiajuzephiniensis* Dekhtyar, 1993	0.46	27
34	*Difflugiaoblonga* Ehrenberg, 1838	0.01	3
35	*Difflugiaparva* (Thomas, 1954) Ogden, 1983	1.12	51
36	*Difflugiapristis* Penard, 1902	0.24	8
37	*Difflugiapulex* Penard, 1890	0.16	12
38	*Difflugiapyriformis* Perty, 1849	0.01	2
39	*Difflugiaurceolata* Carter, 1864	0.01	2
40	*Euglyphaacanthophora* Ehrenberg, 1841	0.01	3
41	*Euglyphaciliata* Ehrenberg, 1848	3.87	173
42	*Euglyphaciliataglabra* Wailes, 1915	0.94	49
43	*Euglyphacristata* Leidy, 1874	0.03	9
44	*Euglyphacristatadecora* Jung, 1942	0.02	4
45	*Euglyphalaevis* Ehrenberg, 1845	2.52	131
46	*Euglyphastrigosa* (Ehrenberg, 1848) Leidy, 1878	0.15	11
47	*Euglyphastrigosaglabra* Wailes, 1898	0.01	1
48	*Euglyphastrigosaheterospina* Wailes, 1912	0.11	4
49	*Euglyphatuberculata* Dujardin, 1841	0.05	12
50	*Galeriporaarenaria* (Greeff, 1866) González-Miguéns et al., 2021	7.28	127
51	*Galeriporaarenariacompressa* (Chardez, 1957) González-Miguéns et al., 2021	0.06	5
52	*Galeriporaarenariasphagnicola* (Deflandre, 1928) González-Miguéns et al., 2021	0.07	8
53	*Galeriporaartocrea* (Leidy, 1876) González-Miguéns et al., 2021	0.41	58
54	*Galeriporacatinus* (Penard, 1890) González-Miguéns et al., 2021	2.36	60
55	*Galeriporadiscoides* (Ehrenberg, 1871) González-Miguéns et al., 2021	0.21	20
56	*Galeriporamegastoma* (Penard, 1902) González-Miguéns et al., 2021	0.01	1
57	*Galeriporapolyporaundulata* (Decloitre, 1976) González-Miguéns et al., 2021	0.01	3
58	*Gibbocarinagaleata* (Penard, 1890) Kosakyan et al., 2016	0.09	4
59	*Heleoperapetricola* Leidy, 1879	1.09	36
60	*Heleoperasphagni* Leidy, 1874	6.83	124
61	*Heleoperasylvatica* Penard, 1890	0.07	12
62	*Hyalospheniaelegans* Leidy, 1874	5.71	136
63	*Hyalospheniapapilio* Leidy, 1874	11.74	185
64	*Lesquereusiaepistomium* Penard, 1902	0.04	4
65	*Lesquereusiainequalis* Cash et Hopkinson, 1909	0.00	1
66	*Lesquereusiaspiralis* Ehrenberg, 1840	0.02	4
67	*Microchlamyspatella* (Claparède et Lachmann, 1859) Cockerell, 1911	0.12	3
68	*Nebelacollaris* (Ehrenberg, 1848) sensu Kosakyan et Gomaa, 2013	0.46	30
69	*Nebelatincta* (Leidy, 1879) Awerintzew, 1906	7.00	186
70	*Netzeliaoviformis* (Cash, 1909) Ogden, 1979	0.02	3
71	*Netzeliatuberculata* Wallich, 1864	0.10	10
72	*Phryganellaacropodia* (Hertwig et Lesser, 1874) Hopkinson, 1909	0.32	30
73	*Phryganellahemisphaerica* Penard, 1902	6.74	118
74	*Physochilatenella* (Penard, 1893) Jung, 1942	7.49	130
75	*Placocistaglabra* Penard, 1905	0.00	2
76	*Placocistalens* Penard, 1899	0.00	1
77	*Pseudodifflugiagracilis* Schlumberger, 1845	0.01	1
78	*Scutiglyphascutigera* (Penard, 1911) Foissner et Schiller, 2001	0.01	1
79	*Sphenoderiafissirostris* Penard, 1890	0.02	4
80	*Sphenoderialenta* Schlumberger, 1845	0.03	3
81	*Tracheleuglyphadentata* (Vejdovsky, 1882) Deflandre, 1928	0.03	4
82	*Trigonopyxisarcula* Penard, 1912	0.44	46
83	*Trigonopyxisminuta* Schönborn et Peschke, 1988	0.21	11
84	*Trinemacomplanatum* Penard, 1890	0.18	15
85	*Trinemaenchelys* Ehrenberg, 1838	0.48	28
86	*Trinemalineare* Penard, 1890	0.59	33

**Table 3.** Environmental variables represented in the dataset.

**Environmental variable**	**Measurement unit**	**Values range**
oxidising and reducing potential (redox)	mkSim/cm	-(103)–290
total mineralisation (tds)	mg/dm^3^	19–94
substrate temperature	°C	13–28
acidity (pH)	pH value	3.4–5.6
substrate wetness	%	90.1–99.51
water table depth (WTD)	cm	0–35
